# The impact of multiple chronic diseases on hospitalizations for ambulatory care sensitive conditions

**DOI:** 10.1186/s12913-016-1584-2

**Published:** 2016-08-04

**Authors:** Inês Dantas, Rui Santana, João Sarmento, Pedro Aguiar

**Affiliations:** 1 National School of Public Health (ENSP), Universidade NOVA de Lisboa, Av. Padre Cruz, 1600-560 Lisbon, Portugal; 2 Department of Health Policy and Systems Management, National School of Public Health (ENSP), Universidade NOVA de Lisboa, Av. Padre Cruz, 1600-560 Lisbon, Portugal; 3 Public Health Research Centre (PHRC), National School of Public Health (ENSP), Universidade NOVA de Lisboa, Av. Padre Cruz, 1600-560 Lisbon, Portugal; 4 Department of Strategies in Health, National School of Public Health (ENSP), Universidade NOVA de Lisboa, Av. Padre Cruz, 1600-560 Lisbon, Portugal

**Keywords:** Avoidable hospitalizations, Ambulatory care sensitive conditions, Chronic conditions

## Abstract

**Background:**

The high financial burden of avoidable hospitalizations has led to an increase of the study of hospitalizations for ambulatory care sensitive conditions (ACSC). There is limited information on the impact of secondary diagnoses on these hospitalizations, although patients’ social and demographic characteristics, as well as the coexistence of multiple diseases are often identified in the literature as risk factors for avoidable hospitalizations. This study explores the impact of chronic conditions on the likelihood of hospitalizations for ACSC.

**Methods:**

Data were extracted from the Portuguese hospital discharge database. Avoidable hospitalizations were identified according to the Canadian Institute for Healthcare Information, and chronic conditions were identified according to criteria set by the Agency for Healthcare Research and Quality. A retrospective study analysing all patients hospitalized for an ACSC and all patients hospitalized for non-ACSC was made, using multiple logistic regression models to identify the impact of chronic conditions on the risk of admission.

**Results:**

The risk of an avoidable hospitalization increases by a factor of 1.35 (95 % CI [1.34;1.35]) for each additional chronic condition, and 1.55 (95 % CI [1.55;1.56]) for each additional body system affected. The respiratory and circulatory systems have the most impact on the risk of ACSC, increasing the risk by 8.72 (95 % CI [8.58;8.86]) and 3.01 (95 % CI [2.95;3.06]), respectively.

**Conclusions:**

The number of chronic conditions and the body systems affected increase the risk of hospital admissions for ACSC.

## Background

Ambulatory care sensitive conditions (ACSC) are conditions for which earlier and more effective ambulatory care can avoid or decrease the rate of hospitalizations [1]. The potential to reduce avoidable hospitalizations associated with these conditions has attracted the interest of healthcare managers, researchers and policy makers in several countries including the United States [2], Canada [3], Australia [4], United Kingdom [5], Spain [6], and Portugal [7].

ACSC are frequently referred to in literature as an indicator of access to care, with the goal of raising awareness of the importance of integrating care as a priority in health policies. The study of ACSC is even more important in the face of current socio-demographic and epidemiological trends, such as aging of the population and living longer with chronic conditions [8], which pose challenges to the needs and expectations of healthcare consumers.

In the United Sates, 65 % of people aged 65 years and older have at least two chronic conditions [9] and 32 % of the hospitalizations are considered to be preventable [10]. Moreover, the likelihood of an ACSC hospitalization for patients with more than one chronic condition is greater than 25 % [11]. This can be partly explained by the under treatment of an unrelated chronic condition [12].

Wolff et al. (2002) studied the prevalence and complications associated with multiple chronic conditions in the Medicare beneficiaries in the United States, observing that the odds of being hospitalized due to an ACSC is 7.5 and 98.5 times greater for patients with at least one and at least four chronic conditions, respectively, when compared to patients without chronic conditions [9].

The relationship between ACSC and the presence of chronic illness is complex in nature, as patients often have multiple chronic conditions. An examination of principal diagnoses and specific combinations of non-principal diagnoses reported in earlier studies show varying levels of risk for an ACSC hospitalization. For example, patients with neurological disorders are 2.6 times more likely to be hospitalized due to an ACSC than patients without neurological disorders [13]; type II diabetes patients, also have a higher probability of an ACSC hospitalization and 96 % have a second chronic condition [14]; and heart failure patients have a 95 % probability of a non-cardiac comorbidity that complicates the original condition [10].

ACSC hospitalizations are proving valuable in measuring access to care and their use as a measure is being adopted by healthcare systems and providers. Combined with the lack of knowledge of the impact of non-principal diagnoses in such hospitalizations, there is a clear need to study how the number of chronic conditions and the body systems they belong to play into ACSC hospitalizations.

## Methods

### Data collection and sampling

We obtained the data used in this study from the Central Administration of the Health System database, which includes patient-level data for all inpatient admissions in mainland Portuguese public hospitals. We considered the available data from 2008 to 2012, as it was the most up to date data set covering five continuous years of data at the start of this study.

The database includes several variables from inpatient admission records including: patient identifier (anonymized), gender, date of birth, principal and secondary diagnoses (using ICD-9-CM), Major Disease Categories of Diagnosis Related Groups (DRG) classification (AP-DRG v27), DRG type (surgical or medical), admission type (emergency or elective), and length of stay, amongst others.

From the original database we excluded the following records:

- Death before discharge;
- Hospitalization length of stay of 0 or more than 39 days (patients with the selected length of stay cover 99 % of the ACSC population);
- Patients less than 36 years old or more than 75 years old (patients with the selected age cover 80 % of the ACSC population).

The final sample included 1,975,542 hospitalizations, accounting for 22 % of the episodes in the original database.

### Identification of ACSC and chronic conditions

We evaluated each hospitalization to identify whether the principal diagnosis was an ambulatory care sensitive condition, using the Canadian Institute for Healthcare Information’s methodology [15]. This method identifies ACSC through ICD-9-CM codes, and groups ACSC into seven categories: grand mal status and other epileptic convulsions, chronic obstructive pulmonary diseases (COPD), asthma, diabetes, heart failure and pulmonary edema (HFPE), hypertension (HTN), and angina.

To identify chronic diseases and the respective body systems, we used the Chronic Condition Indicator methodology, developed as part of the Agency for Healthcare Research and Quality’s Healthcare Cost and Utilization Project [16]. We flagged each diagnosis as a chronic or non-chronic condition, and we identified the body system the chronic condition belongs to – from a predefined list of 18 body systems. Body systems analysis enabled us to study the presence of chronic conditions in additional body systems, identifying only conditions that are distinct from each other. This resulted in the following variables:

- Chronic conditions, which identifies whether the diagnosis is a chronic condition;
- Body systems, which identifies the body system to which the chronic condition relates to;
- Number of chronic conditions for a given patient;
- Number of body systems in which the patient has chronic conditions.

### Statistical analysis

We characterized the sample using descriptive statistics taking into consideration gender, age, admission type, discharge status, length of stay, and DRG type. The study population was divided into two groups: hospitalizations for an ACSC and non-ACSC hospitalizations. We validated the differences between the samples using the Student’s t test and the chi-square test.

We used multiple logistic regressions to evaluate the impact of the non-principal diagnoses in ACSC hospitalizations, adjusted for age and gender. We considered a 95 % confidence interval, and tests with a *p*-value <0.05 were considered to be statistically significant.

## Results

### Study population

The sample had a slightly higher proportion of women (51.5 %) and a concentration in older age groups. Emergency admissions accounted for 50.7 % of the hospitalizations and 95.6 % of the patients were discharged to home. The sample was almost evenly distributed by DRG type, with 49.9 % medical and 50.1 % surgical hospitalizations. The full characterization of the study population is in Table 1.

**Table 1:** Descriptive characteristics comparison of the study’s population and sub-groups

	All hospitalizations		Non-ACSC Hospitalizations		Hospitalizations for ACSC		*p*
Total cases	1 975 542		1 886 302		89 240		
Gender							<0.001
Male	958 003	(48.5 %)	906 014	(48 %)	51 989	(58.3 %)	
Age group							<0.001
36 to 40 years	210 685	(10.7 %)	207 653	(11 %)	3 032	(3.4 %)	
41 to 45 years	184 038	(9.3 %)	180 100	(9.5 %)	3 938	(4.4 %)	
46 to 50 years	205 990	(10.4 %)	200 420	(10.6 %)	5 570	(6.2 %)	
51 to 55 years	227 045	(11.5 %)	219 142	(11.6 %)	7 903	(8.9 %)	
56 to 60 years	250 149	(12.7 %)	239 814	(12.7 %)	10 335	(11.6 %)	
61 to 65 years	271 717	(13.8 %)	258 122	(13.7 %)	13 595	(15.2 %)	
66 to 70 years	290 768	(14.7 %)	272 663	(14.5 %)	18 105	(20.3 %)	
71 to 75 years	335 150	(17 %)	308 388	(16.3 %)	26 762	(30 %)	
Admission type							<0.001
Elective	892 426	(45.2 %)	881 037	(46.7 %)	11 389	(12.8 %)	
Emergency	1 001 108	(50.7 %)	923 298	(48.9 %)	77 810	(87.2 %)	
Others	82 008	(4.2 %)	81 967	(4.3 %)	41	(0 %)	
Average length of stay (days)	6.55		6.50		7.71		0.021
Type of DRG							<0.001
Surgical	989 623	(50.1 %)	987 514	(52.4 %)	2 109	(2.4 %)	
Medical	985 073	(49.9 %)	897 943	(47.6 %)	87 130	(97.6 %)	
Other	846	(0 %)	845	(0 %)	1	(0 %)	
Average of DRG weight	0.9853		0.9853		0.9858		0.003
Discharge status							<0.001
Home	1 887 677	(95.6 %)	1 802 660	(95.6 %)	85 017	(95.3 %)	
Another hospital	61 246	(3.1 %)	58 141	(3.1 %)	3 105	(3.5 %)	
Others	26 619	(1.3 %)	25 501	(1.4 %)	1 118	(1.3 %)	

*Note*: Table shows % within group

Of all hospitalizations, 4.5 % were attributed to ambulatory care sensitive conditions. Among these hospitalizations, 28.5 % were attributed to heart failure and pulmonary edema and 25.8 % were attributed to COPD. In the 71 to 75 year old age group, the hospitalization rate for ACSC was 8.0 %, almost double the rate for the overall sample.

When comparing ACSC hospitalizations with non-ACSC hospitalizations, we observed a higher frequency of males (58.3 %, *p* < 0.001) and older patients (*p* < 0.001). For ACSC hospitalizations the admission type is emergency in 87.2 % of cases and the DRG is medical in most cases (97.6 %). The average length of stay increases from 6.50 days to 7.71 (*p* = 0.021) and the DRG weight rises from 0.9853 to 0.9858 (*p* < 0.001).

### Chronic conditions in the population

Just over half (51.7 %) of all patients had at least two chronic conditions and 20.3 % had at least four chronic conditions. These rates increased to 88.0 % and 50.4 % respectively for the ACSC hospitalizations group. The average number of chronic conditions in the ACSC group was 3.93, twice the average of the non-ACSC group (*p* = 0.008). Moreover, the ACSC group chronic conditions affected an average of 2.5 body systems, compared to 1.49 body systems in the non-ACSC group (*p* = 0.004). These results are in Table 2.

**Table 2:** Number of chronic conditions and body systems affected in the study’s population and sub-groups

	All hospitalizations	Non-ACSC hospitalizations	Hospitalizations for ACSC	*p*
Number of hospitalizations	1 975 542	1 886 302	89 240	
Average number of chronic conditions	2.07	1.98	3.93	0.008
Number of chronic conditions (% of column)				
At least 1	76.3	75.2	100.0	
At least 2	51.7	50.0	88.0	
At least 3	33.4	31.7	69.7	
At least 4	20.3	18.9	50.4	
Average number of body systems	1.53	1.49	2.50	0.004
Number of additional body systems (% of column)				
At least 1	76.3	75.2	100.0	
At least 2	44.9	43.3	78.4	
At least 3	21.0	19.9	43.7	
At least 4	7.7	7.2	18.6	

To better understand how chronic conditions relate to each specific ACSC, we further studied four sub-populations, corresponding to the hospitalizations in which the principal diagnosis belonged to one of the four most frequent body systems in ACSC hospitalizations: endocrine, nutritional, metabolic diseases, and immunity disorders; diseases of the nervous system and sense organs; diseases of the circulatory system; and diseases of the respiratory system. In these sub-populations, more than 54.0 % of hospitalizations have additional chronic conditions. This value rose to 89.4 % when the principal diagnosis is a disease of the circulatory system, reflecting the increased disease accumulation in these cases. More than 8.7 % of this population has chronic conditions in at least four different body systems. Regardless of the chronic condition that is responsible for the hospitalization, most patients accumulate chronic conditions in the following body systems: endocrine, nutritional, metabolic diseases, and immunity disorders, diseases of the circulatory system, and diseases of the respiratory system (Table 3).

**Table 3:** Chronic conditions and body systems in the admissions for the most common ACSC

	Hospitalizations with a primary diagnosis of				
	Endocrine, nutritional, and metabolic diseases and immunity disorders	Diseases of the nervous system and sense organs	Diseases of the circulatory system	Diseases of the respiratory system	*p*
Number of hospitalizations	61 108	54 575	263 642	36180	
Average number of chronic conditions	3.12	2.23	3.91	3.47	<0.001
Number of chronic conditions (% of column)					
At least 2	74.2	54.0	89.4	79.9	
At least 3	51.3	32.0	72.0	60.5	
At least 4	33.5	17.8	51.6	41.8	
Average number of body systems	2.24	1.84	2.19	2.55	<0.001
Additional body systems (% of column)					
At least 1	68.1	47.9	72.4	73.9	
At least 2	34.8	24.8	31.7	47.2	
At least 3	14.6	8.7	10.6	22.0	
Most common body system					
Rank 1	Circulatory (45.0 %)	Circulatory (25.9 %)	Endocrine (54.3 %)	Circulatory (45.7 %)	
Rank 2	Nervous (17.3 %)	Endocrine (21.9 %)	Mental (17.1 %)	Endocrine (33.7 %)	
Rank 3	Mental (12.7 %)	Mental (14.3 %)	Nervous (9.3 %)	Mental (21.3 %)	

### Impact of chronic conditions in ACSC hospitalizations

Being a male and in a higher age group increased the risk of an ACSC hospitalization in every model.

For each chronic condition a patient had, the risk of an ACSC hospitalization increased 1.35 times (Table 4). The risk of an ACSC hospitalization for patients with at least four chronic diseases was 3.29 times higher than that of a patient without any chronic conditions. The number of body systems affected also increased the risk of an ACSC hospitalization, raising the risk by 1.55 for each additional body system (Table 5).

**Table 4:** Logistic regression for ACSC hospitalizations using the number of chronic conditions as the explanatory variable

Independent variable	Sig	Odds ratio	95 % CI
Number of chronic conditions	<0.001	1.35	(1.34-1.35)
Age groups			
41 to 45 years	<0.001	.34	(0.32-0.35)
46 to 50 years	<0.001	.42	(0.41-0.43)
51 to 55 years	<0.001	.47	(0.46-0.49)
56 to 60 years	<0.001	.56	(0.54-0.57)
61 to 65 years	<0.001	.60	(0.59-0.61)
66 to 70 years	<0.001	.68	(0.67-0.70)
71 to 75 years	<0.001	.80	(0.79-0.82)
Gender	<0.001	1.23	(1.21-1.24)
Predictive accuracy of the model: 95.4 %			
Omnibus tests: *p* < 0.001			
Area under the curve: 0.762

**Table 5:** Logistic regression for ACSC hospitalizations using the number of body systems as the explanatory variable

Independent variable	Sig	Odds ratio	95 % CI
Number of body systems	<0.001	1.55	(1.55-1.56)
Age groups			
41 to 45 years	<0.001	.31	(0.30-0.33)
46 to 50 years	<0.001	.39	(0.38-0.40)
51 to 55 years	<0.001	.44	(0.43-0.45)
56 to 60 years	<0.001	.52	(0.51-0.53)
61 to 65 years	<0.001	.57	(0.56-0.59)
66 to 70 years	<0.001	.66	(0.64-0.67)
71 to 75 years	<0.001	.79	(0.78-0.81)
Gender	<0.001	1.27	(1.25-1.29)
Predictive accuracy of the model: 95.5 %			
Omnibus tests: *p* < 0.001			
Area under the curve: 0.735			

The body systems in which having chronic conditions had a greater impact on the risk of an ACSC hospitalization were: diseases of the respiratory system (8.72, 95 % IC *p* < 0.001), diseases of the circulatory system (3.01, 95 % IC *p* < 0.001), endocrine, nutritional and metabolic diseases, and immunity disorders (1.64, 95 % IC *p* < 0.001), and diseases of the nervous system and sense organs (1.58, 95 % IC *p* < 0.001). If a patient had both a disease of the respiratory system and a disease of the circulatory system, the risk of an ACSC hospitalization was 26.19 times greater. Accumulating chronic conditions in all four body systems increased the risk of an ACSC hospitalization by 67.91.

## Discussion

The identification of hospitalizations for ambulatory care sensitive conditions does not commonly take into consideration multiple chronic conditions. However, in this study we determined that not only is the risk of an ACSC hospitalization higher for patients with multiple chronic conditions, but also that patients hospitalized for an ACSC have more chronic diseases and in more body systems than those hospitalized for a non-ACSC.

### Study population

Our population characteristics were similar to the population in the literature reviewed. In the context of the present study, the most relevant results in literature are the increased prevalence of chronic conditions with age [9, 14] and the concentration of ACSC hospitalizations in the male and older population [9, 17]. The most frequent ACSC found were heart failure and pulmonary edema, the same as reported by Sarmento [18].

The rate of ACSC hospitalizations in our sample was 4.5 %, which rose to 7.9 % in the population over 65 years old, values similar to those obtained by Niti and Ng [19] and results obtained in Portugal [17, 20]. The calculated rates are lower than other similar studies probably because of the higher percentage of elective hospitalizations in our study, which were nearly half of the population studied.

### Chronic conditions in the population

Across all the hospitalizations in our sample we found that 76.3 % had at least one chronic condition, 51.7 % had at least two chronic conditions, and 20.3 % had at least four chronic conditions. These results are similar to those described by Ajmera [11] and Wolff, Starfield, and Anderson [9].

Hospitalizations for diseases of the circulatory or respiratory systems are associated with a higher burden of chronic disease. For hospitalizations with a chronic condition in the circulatory or respiratory systems as the principal diagnosis, we found that 89.4 % and 79.9 % of the patients, respectively, had at least two chronic conditions and 51.6 % and 41.8 %, respectively, had at least four chronic conditions.

When considering only ACSC hospitalizations, we found that the average number of both chronic conditions and body systems was higher than in non-ACSC hospitalizations. In fact, we observed an average of 3.93 chronic conditions in ACSC hospitalizations and only 1.98 chronic conditions in non-ACSC hospitalizations. When looking at body systems, we observed an average of 2.50 for ACSC hospitalizations and 1.49 for non-ACSC hospitalizations. Ajmera and Wolff have reported similar findings [9, 11].

### Impact of non-principal diagnoses in ACSC hospitalizations

When studying the likelihood of being hospitalized for an ACSC, we found that the risk of such an event increases by a factor of 1.35 for each additional chronic condition and 1.55 for each additional body system.

Considering the body systems themselves, we found that having a chronic condition in the circulatory system or in the respiratory system increases the risk of an ACSC hospitalization by a factor of 8.72 and 3.01, respectively. There are similar results in the literature [21, 22], although earllier studies focused on patients with specific conditions. Balogh et al. [23], for example, identified a 2.6 times higher risk of hospitalization in patients with mental disorders.

The fact that the risk of an ACSC hospitalization increases with the number of chronic conditions can be explained by the patients’ difficulty in managing the disease burden, particularly managing and adhering to various and complex therapeutic regimens [14]. This increased risk also suggests the challenges healthcare providers face with interdisciplinary and multidisciplinary care.

### Recommendations

Using ACSC hospitalizations as a measure of access to care requires a good understanding of as many influencing factors as possible. Since the rates of hospitalization increase with both the number of chronic conditions and the number of body systems, these variables must be taken into account in the development of risk models for ACSC hospitalization indicators. Doing so will produce more meaningful findings when evaluating ACSC hospitalizations in the future, when performing countrywide or regional analyses.

Our findings indicate that chronic condition patterns are associated with varying risks of hospitalization. Therefore, individuals should be identified and targeted for specific prevention, treatment, and follow-up based on their chronic condition patterns, which represent different risks of hospitalization. Health policies should also consider strategies to stratify the population according to their chronic disease burden and risk of hospitalization, in order to plan appropriate care delivery and allocation of healthcare resources.

Interventions to reduce the rate of hospitalizations due to ACSC should also take this studies’ findings in consideration, targeting patients at higher risk through better health promotion, using alternative clinical pathways, or enabling self-care. Integrated care should leverage technology to make more and better information available to clinicians –regarding both the patient’s health status and condition-specific best practices – thereby reducing the current number of ACSC hospitalizations.

### Study limitations

From a methodology point of view, the quality of the data used is the main limitation. There are no known studies in Portugal that assure the accuracy or completeness of documented diagnoses. This is particularly important since the database is used for reimbursement purposes and data might not be documented in a way that is preferable for clinical studies. Most studies using the same database share this concern [24].

We could have used patients instead of hospitalizations as the unit of analysis. Doing so would provide more detail to characterize the secondary diagnosis but would reduce the burden of disease in the hospitalizations. Similar studies also consider hospitalizations instead of patients. Finally, the fact that we studied diagnoses in the context of an acute episode of care, and not longitudinally, did not allow us to explore a possible relationship of causality between morbidity and ACSC hospitalizations.

### Further research

This study quantifies the effect of comorbidities in ACSC hospitalizations and the impact of multiple chronic conditions in these hospitalizations. This research would be enhanced by further exploring the onset and duration of chronic conditions, and probing potential causality between specific conditions and ACSC hospitalizations.

In addition, since it is clear that there are several variables influencing ACSC hospitalizations, it would be interesting to bundle the impact of chronic conditions with additional variables in order to build a model with increased power for predicting the risk of hospitalization due to ACSC.

## Conclusion

This study adds to the knowledge on the impact of the secondary diagnosis in preventable hospitalizations. The risk of a preventable hospitalization increases by a factor of 1.35 for each additional chronic condition and 1.55 for each additional body system. The body systems with the greatest impact in the risk of a preventable hospitalization are the respiratory and circulatory systems, the risk increasing by factors of 8.72 and 3.01, respectively.

These results point to the need to focus ambulatory setting interventions on patients with multiple chronic conditions, especially through continuous actions at a disease management level for the different chronic diseases, seeking to minimize the impact of chronic conditions in hospitalizations for ACSC.

Due to the increased prevalence of chronic conditions and high cost associated with treating chronic patients, further studying secondary diagnosis in preventable hospitalizations is critical to identify strategies that can minimize the impact of such hospitalizations on the Portuguese National Health System.

## Abbreviations

ACSC, ambulatory care sensitive conditions; COPD, chronic obstructive pulmonary diseases; DRG, diagnosis related groups; HFPE, heart failure and pulmonary edema; HTN, hypertension
